# Effects of green tea use on the metabolic profile of postmenopausal women: systematic review and meta-analysis

**DOI:** 10.1007/s00394-026-04005-8

**Published:** 2026-06-02

**Authors:** Isabella Heloísa Rodrigues Zago, Laura Colonetti, Eduarda Letícia Balbinot, Igor Specht Taschetto, Antonio José Grande, Maria Inês da Rosa, Tamy Colonetti

**Affiliations:** 1 School of Medicine, Universidade do Extremo Sul Catarinense (UNESC), Criciúma, SC Brazil; 2 Laboratory of Translational Biomedicine, Universidade do Extremo Sul Catarinense, Criciúma, SC Brazil; 3 Laboratory of evidence-based practice, Universidade Estadual de Mato Grosso do Sul, Campo Grande, MS Brazil

**Keywords:** Postmenopause, Green tea, Metabolic syndrome

## Abstract

**Purpose:**

Among the main consequences of menopause are changes in body weight, lipid and metabolic profiles, as well as an increased risk of cardiovascular disease. This study aimed to analyze the effects of green tea consumption on the metabolic profile of postmenopausal women.

**Methods:**

This is a systematic review of randomized controlled trials (RCTs) evaluating the effects of green tea compared with placebo in postmenopausal women. The search was conducted in the following electronic databases: MEDLINE via PubMed, EMBASE via Elsevier, the Cochrane Library, LILACS via BVS, and Web of Science, using the terms “Tea,” “Green tea extract,” “Metabolic profile”, “Menopause,” and “Postmenopause” (MeSH descriptors and synonyms). The outcomes listed in the study protocol were: weight, body mass index (BMI), body composition, lipid profile, and blood glucose.

**Results:**

Data were collected from seven RCTs that met the selection criteria. The meta-analysis showed that green tea reduced total cholesterol levels in postmenopausal women (mean difference [MD]: −7.03; 95% confidence interval [CI]: −13.24 to − 0.82; *p* = 0.03; I² = 0%; four studies; 1,109 participants; low-quality evidence). However, no statistically significant effects were observed for the other evaluated outcomes.

**Conclusion:**

This meta-analysis demonstrated that green tea resulted in reductions in total cholesterol levels in postmenopausal women. However, further high-quality randomized controlled trials with standardized dosage, formulation, and duration are needed to confirm these findings and support the safe clinical use of green tea as an adjunctive strategy. High-dose concentrated extracts should be used with caution, and hepatic function monitoring may be warranted.

**Supplementary Information:**

The online version contains supplementary material available at https://doi.org/10.1007/s00394-026-04005-8.

## Introduction

Menopause is retrospectively defined as the absence of menstruation for 12 consecutive months and marks the end of female reproductive function. This transition results from the progressive depletion of ovarian follicles, leading to reduced estrogen and progesterone secretion [1]. Most women experience menopause between 49 and 52 years of age. With increasing life expectancy, women are expected to spend at least 40% of their lives in the postmenopausal period [2].

The postmenopausal period is marked by profound metabolic alterations driven primarily by estrogen deficiency. These changes are associated with an increased risk of several chronic conditions, including cardiovascular disease, hormone-sensitive breast cancer, insulin resistance, type 2 diabetes mellitus (T2DM), and osteoporosis. Moreover, hormonal fluctuations during perimenopause and menopause are responsible for a range of characteristic symptoms [3]. Clinically, urinary symptoms and vasomotor manifestations are common and are typically reported as hot flashes and night sweats [4].

Sleep disturbances, emotional lability, depression, and anxiety are also frequently reported during the menopausal transition, along with reduced libido and cognitive decline [5]. In addition, metabolic alterations, such as increased abdominal adiposity, insulin resistance, and dyslipidemia, pose significant challenges during this period, as they are closely associated with changes in body weight and fat distribution [6].

Green tea (Camellia sinensis) has gained considerable attention due to the biological activity of its polyphenols, particularly epigallocatechin-3-gallate (EGCG), which is recognized as its most active compound. EGCG has been shown to modulate key metabolic pathways, including activation of AMP-activated protein kinase (AMPK), increased fatty acid oxidation, inhibition of lipogenesis, and improvement of insulin sensitivity. Additionally, catechins exhibit antioxidant and anti-inflammatory properties that may contribute to cardiometabolic protection [7].

Previous systematic reviews and meta-analyses have demonstrated beneficial effects of green tea on lipid profile and cardiometabolic risk factors in general adult populations. However, these findings cannot be directly extrapolated to postmenopausal women due to the distinct endocrine and metabolic context associated with estrogen deficiency. Furthermore, variability in green tea formulations (extract vs. infusion), caffeine content, and EGCG dosage may differentially influence outcomes in this population. Thus, despite growing evidence on the metabolic effects of green tea, there remains a lack of synthesis focused specifically on postmenopausal women, a population with unique physiological characteristics and increased cardiometabolic risk [8]. Therefore, the aim of the present study was to systematically evaluate the effects of green tea consumption on the metabolic profile of postmenopausal women.

## Methods

### Study design

A systematic review was conducted in accordance with the Preferred Reporting Items for Systematic Reviews and Meta-Analyses (PRISMA) guidelines [9].

### Protocol and registration

As this study is a systematic review, submission to a Research Ethics Committee was not required. However, to formally register the study at the international level, the protocol was registered in the International Prospective Register of Systematic Reviews (PROSPERO) under registration number CRD420250651988.

### PICO

Evidence-based practice (EBP) recommends that clinical questions arising from care, teaching, or research be structured using the PICO framework, an acronym for Population, Intervention, Comparison, and Outcomes.

Accordingly, studies were included if they met the eligibility criteria defined by the PICO framework of the present review:

- *Population*: Postmenopausal women;
- *Intervention*: Green tea consumption (extract or infusion);
- *Comparison*: Placebo;
- *Outcomes*: Body weight, body mass index (BMI), body composition, fat mass, waist circumference, total cholesterol, high-density lipoprotein (HDL), low-density lipoprotein (LDL), triglycerides, adiponectin, leptin, fasting glucose, serum insulin, and the Homeostatic Model Assessment of Insulin Resistance (HOMA-IR);
- *Study design*: Randomized controlled trials.

### Eligibility criteria

#### Inclusion criteria

Randomized controlled trials (RCTs) were included if they evaluated changes in body weight and/or body composition or BMI, lipid profile, glycemia, and other metabolic outcomes following green tea consumption in postmenopausal women compared with control participants.

#### Exclusion criteria

Studies were excluded if participants presented conditions that could influence metabolic outcomes, including thyroid dysfunction, diabetes mellitus, renal disease, cancer, or a history of hepatic or gallbladder disorders. Additionally, studies were excluded if participants reported any habitual consumption of green tea prior to the intervention, regardless of dose or frequency, in order to avoid potential baseline exposure bias.

### Search strategy

A comprehensive search strategy was developed using Medical Subject Headings (MeSH) terms and their synonyms, including: “Tea”, “Green tea extract”, “Metabolic profile”, “Menopause”, and “Postmenopause”. These terms were combined using Boolean operators (“AND” and “OR”) to maximize sensitivity. The search was conducted in the following electronic databases: MEDLINE (via PubMed), Embase, Web of Science, Cochrane Library, and LILACS. The last search was performed on November 2025. No language restrictions were applied, and only studies conducted in humans were included. In addition, the reference lists of included studies were manually screened to identify potentially relevant articles.

The MEDLINE (via PubMed) search strategy was as follows:

(“Tea“[Mesh] OR “Green Tea“[Mesh] OR “Camellia sinensis“[All Fields] OR “green tea“[All Fields] OR “green tea extract“[All Fields] OR “green tea catechins“[All Fields] OR “epigallocatechin gallate“[All Fields] OR “EGCG“[All Fields] OR “Polyphenon E“[All Fields])

AND.

(“Menopause“[Mesh] OR “Postmenopause“[Mesh] OR “postmenopausal“[All Fields] OR “postmenopause“[All Fields])

Equivalent search strategies were adapted for Embase, Web of Science, Cochrane Library, and LILACS using appropriate controlled vocabulary and syntax. The full search strategies for all databases are provided in Supplementary Material.

### Study screening and selection

#### Title and abstract screening

After applying the search strategies across the databases, all retrieved records were exported to the Rayyan platform (www.rayyan.qcri.org) for title and abstract screening. Two reviewers (IZ and LC) independently assessed the studies and identified those that met the eligibility criteria. Studies considered potentially eligible for inclusion in the systematic review were selected for full-text assessment. Any disagreements between the reviewers during this process were resolved by consensus or, when necessary, by consultation with a third reviewer (TC).

#### Full-text assessment

For studies selected for full-text review, two reviewers (IZ and LC) independently evaluated whether the articles adequately fulfilled the inclusion criteria. Disagreements were resolved by consultation with a third reviewer (TC).

#### Data extraction

Data extraction from studies included in the systematic review was performed using a standardized data collection form, which included the following information: study characteristics (author, year, country), study objectives, participant characteristics (age, sample size, menopausal diagnostic criteria), study methods, details of the intervention and placebo (including dose, duration, and mode of administration), and outcomes of each included study. This process was conducted independently by two reviewers (IZ and LC).

### Risk of bias assessment

The risk of bias was assessed using the Cochrane Risk of Bias tool, version 2.0 (RoB 2.0) [10]. This tool evaluates potential sources of bias in the assessment of intervention effects across the studies included in the systematic review, namely: bias arising from the randomization process; bias due to deviations from the intended interventions; bias due to missing outcome data; bias in the measurement of the outcome; and bias in the selection of the reported result.

The assessment comprises five domains, which are judged based on signaling questions answered by the reviewers as “Yes”, “Probably yes”, “Probably no”, “No”, or “No information”. The combination of responses within each domain allows classification of the risk of bias through an algorithm into the categories “Low”, “High”, or “Some concerns”. Risk of bias assessment was conducted in duplicate by two independent reviewers, in accordance with the Cochrane Handbook.

### Statistical analysis

Results were presented in tables and figures. Meta-analyses were conducted using Review Manager (RevMan), version 5.4. Continuous outcomes were pooled using mean differences (MD) with 95% confidence intervals (CI). Statistical heterogeneity was assessed using the I² statistic and interpreted according to Cochrane guidelines: 0–30%: low heterogeneity, 30–60%: moderate, 50–90%: substantial, 75–100%: considerable. A fixed-effect model (Mantel–Haenszel) was applied when I² ≤ 40%, while a random-effects model was used when I² > 40%. When data required for meta-analysis were missing or incomplete, attempts were made to contact study authors. If standard deviations were not available, they were estimated from available data (e.g., confidence intervals or standard errors), when possible. Otherwise, the study was excluded from quantitative synthesis and included in the descriptive analysis. Publication bias was not assessed using funnel plots due to the inclusion of fewer than 10 studies per outcome, in accordance with Cochrane recommendations [11].

### Quality of evidence assessment

The quality of evidence was evaluated using the GRADEpro system (www.gradepro.org), in which the overall certainty of evidence is classified as “High”, “Moderate”, “Low”, or “Very low”. As this review included randomized controlled trials, the certainty of evidence initially started as “High” and could be downgraded by one or two levels based on the presence of risk of bias, inconsistency, indirectness, imprecision, or publication bias.

## Results

A total of 1568 records were identified through the search strategy. Of these, 215 records were excluded as duplicates, leaving 1353 studies for title and abstract screening. After title and abstract screening, 1309 records were excluded for not meeting the inclusion criteria, leaving seven studies classified as “possibly eligible” and 44 articles selected for full-text assessment. Five reports could not be retrieved, resulting in 39 studies assessed for eligibility at the full-text level. Of these, five were excluded due to an inappropriate study design, 12 due to a different study population, eight due to different outcomes, one due to a different intervention, one poster abstract, four randomized clinical trial registry records, and one study protocol. Ultimately, seven randomized controlled trials were included in this systematic review [8, 12–17].

**Fig. 1:**
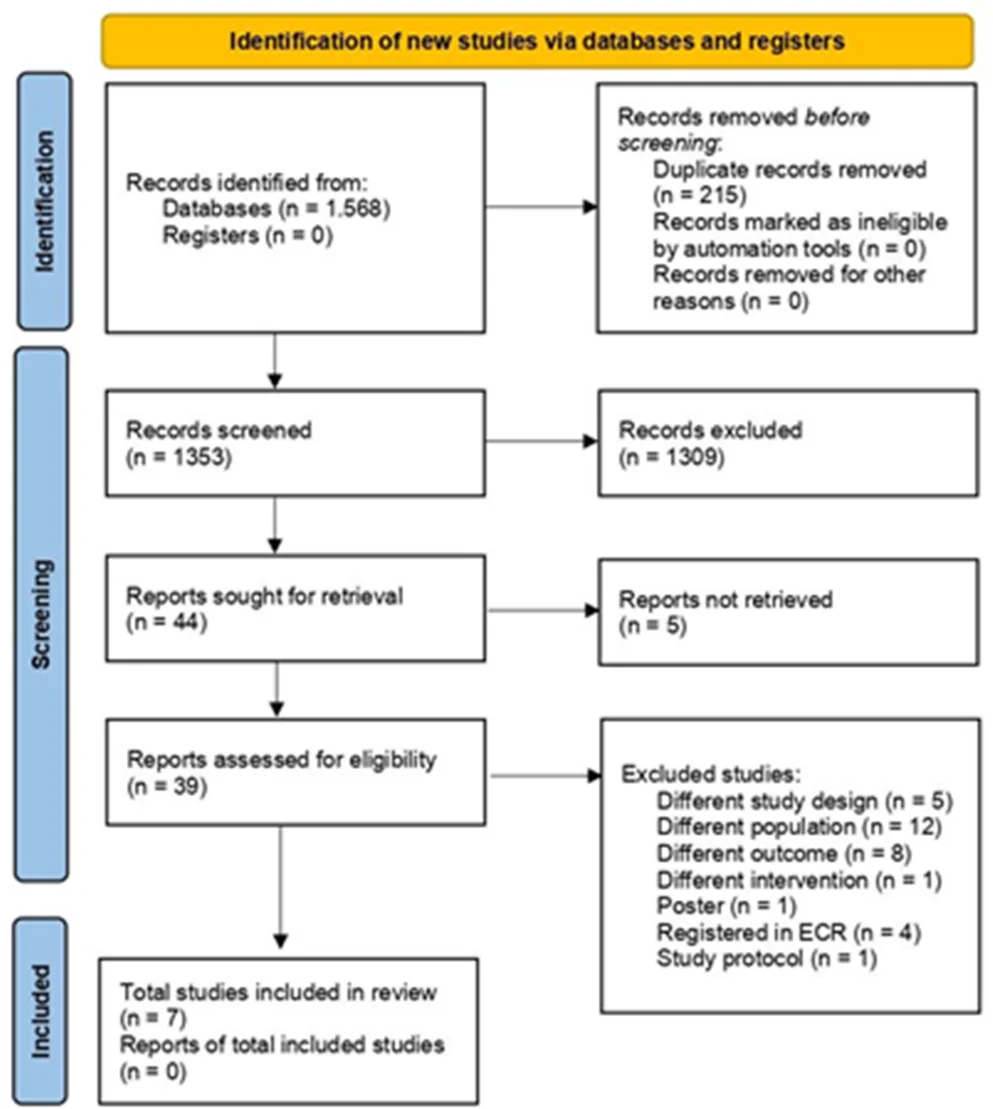
Flow diagram of study selection. *Source*: Prepared by the authors (2026)

The study selection process is presented in the PRISMA flow diagram (Fig. 1).

### Description of included studies

The seven included studies were published between January 2012 and January 2023 and involved a total of 549 participants, all of whom were women, with a mean age ranging from 49 to 67 years. All participants were in the postmenopausal period, which was clinically diagnosed based on temporal criteria (time since last menstruation) and/or hormonal criteria (follicle-stimulating hormone [FSH] levels). The general characteristics of the included studies are summarized in Table 1.

**Table 1:** Characteristics of the included studies. *Source*: Authors, 2026

Author, year (study design, Country)	Age (years)	*N* |intervention/control		Mean age (years) ± SD (intervention/control)	Duration (months)		Type of intervention	Dose/frequency/route of administration	Type of control used	Conclusion
Samavat et al.^8^] (RCT, USA)	50–70	463 / 473	60.02 ± 4.89/ 59.65 ± 5.04			12	Capsules containing a decaffeinated green tea extract (GTE) catechin complex	Decaffeinated GTE was administered for 12 months (1.315 mg of catechins/day; 843 mg of EGCG), in two capsules after breakfast and two after dinner.	Placebo capsules composed mainly of maltodextrin and cellulose.	Supplementation with GTE significantly reduced total cholesterol and LDL, particularly in individuals with elevated baseline levels.
Dostal et al. [12] (RCT, USA)	50–70	117 / 120	60.09 ± 0 0.45/ 60.6 ± 0.47			12	Capsules containing a decaffeinated green tea extract (GTE) catechin complex	Each capsule contained 328 ± 30 mg of catechins (211 ± 11 mg of EGCG), for a total daily intake of 1.315 ± 116 mg of catechins (843 ± 44 mg of EGCG), equivalent to five 240-mL cups of green tea, administered as two capsules in the morning and two in the evening.	Placebo capsules containing 816 mg of maltodextrin, 808 mg of cellulose, and 8 mg of magnesium stearate as a flow agent.	Decaffeinated GTE reduced fasting insulin without altering body weight or hormonal levels; high COMT activity may increase insulin and reduce adiponectin.
Dostal et al. [13] (RCT, USA)	50–70	61 / 60	60.7 ± 0.60/ 60.0 ± 0.65			12	Capsules containing a decaffeinated green tea extract (GTE) catechin complex	Decaffeinated GTE: 1.315 ± 116 mg/day of catechins (843 ± 44 mg of EGCG), equivalent to approximately five 240-mL cups of green tea, administered as two capsules in the morning and two in the evening.	Placebo capsules containing 816 mg of maltodextrin, 808 mg of cellulose, and 8 mg of magnesium stearate as a flow agent.	GTE did not reduce total adiposity or improve BMD, but may benefit the reduction of gynoid fat in women with higher BMI.
Tadayon et al. [14] (RCT, Iran)	45–60	39 / 40	53.7 ± 4.1/ 52.9 ± 3.6			1	Green tea capsules	Green tea extract capsules: 400 mg per capsule (40–47 mg of polyphenols). Taken twice daily, after breakfast and dinner, for 4 weeks.	Placebo capsules containing starch and with an appearance similar to the green tea capsules.	GTE reduced total cholesterol, LDL, and triglycerides in postmenopausal women with mild lipid abnormalities, without significant adverse effects.
Rondanelli et al. [15] (RCT, Italy)	Not reported	14/14	56.92 ± 5.7/ 60.57 ± 7.28			2	Greenselect Phytosome^®^	GSP: 150 mg per tablet; 2 tablets/day (before lunch and before dinner), standardized to ≥ 19% catechins, ≥ 13% EGCG, and ≤ 0.1% caffeine.	Identical capsules containing microcrystalline cellulose, administered following the same instructions as the intervention group.	GSP supplementation improved lipolysis and reduced total, visceral, and abdominal fat in postmenopausal women with overweight or class I obesity.
Takahashi et al. [16] (RCT, Japan)	62–73	11/11	66.6 ± 1.2/ 66.5 ± 0.6			1	Catechin-rich green tea beverage	350 mL/day of green tea, providing 615 mg of total catechins (125.9 mg EGCG) and 77 mg of caffeine, consumed with breakfast for 4 weeks.	350 mL/day of a placebo beverage containing 92 mg of total catechins (15 mg EGCG) and 85.2 mg of caffeine, consumed with breakfast for 4 weeks.	Daily intake of catechin-rich green tea for 4 weeks reduced postprandial glucose and increased serum thioredoxin levels, without altering oxidative stress markers, in healthy postmenopausal women.
Wu et al. [17] (RCT, USA)	Not reported	Group 400 EGCG: 37 Group 800 EGCG: 34/32	Group 400 mg EGCG: 59.6 years ± 6.36 Group 800 mg EGCG: 62.0 years ± 9.42/57.7 years ± 6.29			2	Oral green tea extract supplement (PPE^®^) rich in EGCG.	Each capsule contained 200 mg of EGCG and other catechins; the 800 mg group took 4 capsules/day and the 400 mg group took 2 capsules/day for 2 months.	Placebo capsules contained pregelatinized starch, colloidal silicon dioxide, and magnesium stearate; administered in the same manner as the intervention groups	In postmenopausal women, green tea supplementation (400–800 mg/day for 2 months) reduced LDL and improved glycemic markers.

SD: standard deviation; N: total number of participants; RCT: randomized clinical trial; USA: United States of America; GTE: Green Tea Extract Catechin Complex; LDL: low-density lipoprotein; EGCG: epigallocatechin-3-gallate; COMT: catechol-O-methyltransferase; mL: milliliters; mg: milligrams; BMI: body mass index; tab: tablet; PPE^®^: Polyphenon E^®^; BMD: bone mineral density; GSP: Greenselect^®^ Phytosome^®^, a standardized green tea extract containing ≥ 19% catechins and ≥ 13% EGCG; *p* < 0.05 was considered statistically significant

### Anthropometric outcomes

**Fig. 2:**
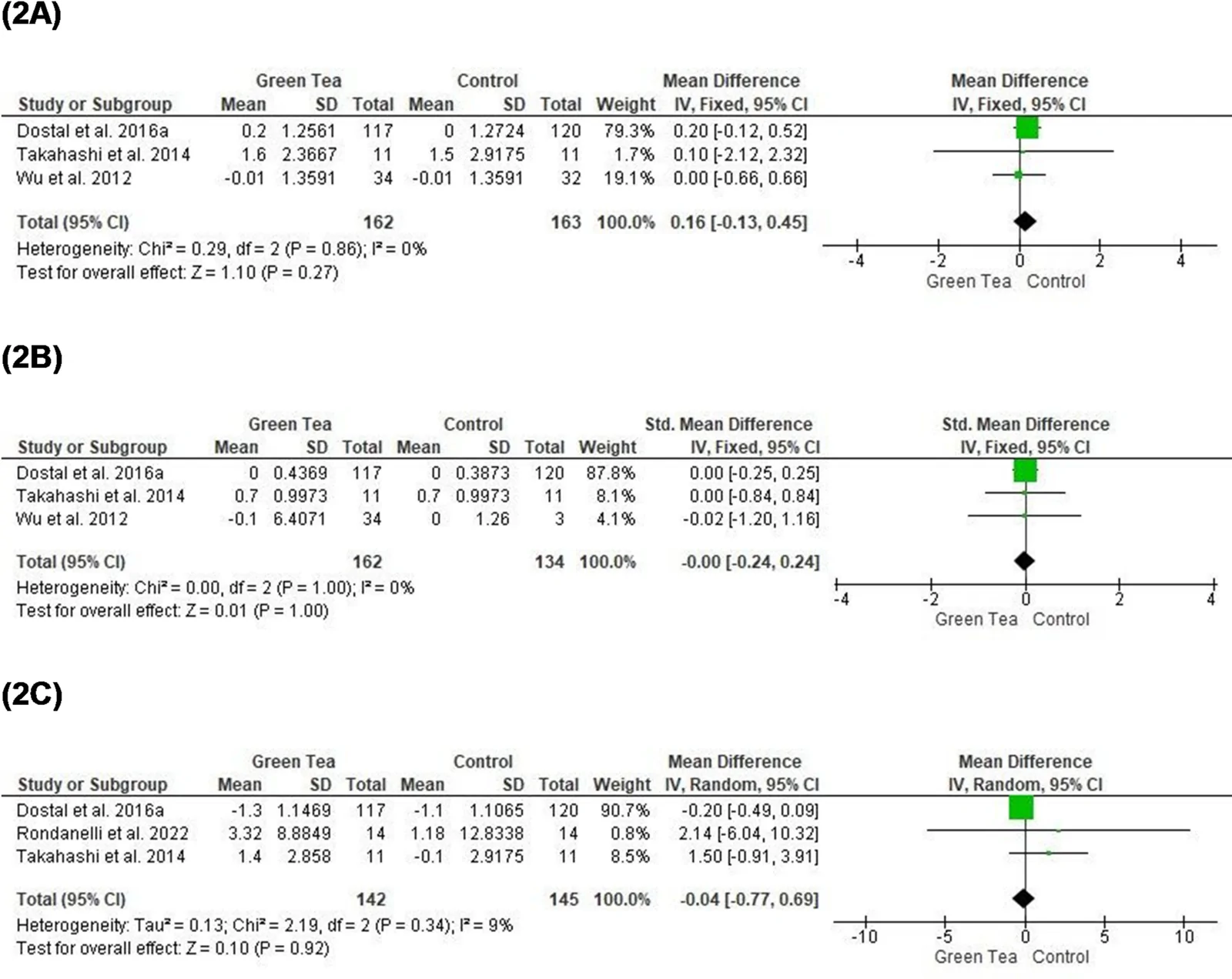
Meta-analysis of anthropometric outcomes: body weight (**2A**), body mass index (BMI) (**2B**), and waist circumference (**2C**). *Source*: Prepared by the authors (2026)

Body weight was assessed in three of the included studies [12, 16, 17], comprising a total of 162 participants in the intervention group and 163 in the control group. For outcome analysis, the change in body weight observed in each group during the intervention period was compared. No statistically significant difference was found between the intervention and control groups (MD: 0.16; 95% CI: −0.13 to 0.45; *p* = 0.27; I² = 0%; three studies; 325 participants; very low certainty of evidence). The meta-analysis of body weight reduction is presented in Fig. 2A.

The same studies also assessed body mass index (BMI) [12, 16, 17]. Similar to body weight, comparison of BMI changes before and after the intervention showed no statistically significant differences between the intervention and control groups (MD: −0.00; 95% CI: −0.24 to 0.24; *p* = 1.00; I² = 0%; three studies; 325 participants; very low certainty of evidence). The meta-analysis of BMI reduction is shown in Fig. 2B.

Waist circumference was evaluated in three of the included studies [12, 15, 16], comprising 142 participants in the green tea group and 145 in the control group. No statistically significant difference was observed in waist circumference reduction between groups (MD: −0.04; 95% CI: −0.77 to 0.69; *p* = 0.92; I² = 0%; three studies; 287 participants; low certainty of evidence). The meta-analysis of this outcome is presented in Fig. 2C.

### Lipid profile

Based on the included studies, differences in the lipid profile of participants were analyzed. Total cholesterol, HDL cholesterol, LDL cholesterol, and triglycerides were evaluated in four studies included in this systematic review [8, 14, 15, 17], comprising a total of 550 participants in the green tea group and 559 in the control group.

Between-group comparisons revealed a statistically significant difference in total cholesterol, indicating a favorable effect of the intervention in participants who received green tea, with a greater reduction compared with the control group (MD: −7.03; 95% CI: −13.24 to − 0.82; *p* = 0.03; I² = 0%; four studies; 1,109 participants; low certainty of evidence). It is important to note that Samavat et al. [8] contributed approximately 75% of the weight in the pooled analysis for total cholesterol. Therefore, although the overall effect was statistically significant, this finding should be interpreted with caution because the pooled estimate was largely influenced by a single large trial.

No statistically significant differences were observed for HDL cholesterol (MD: −1.16; 95% CI: −3.54 to 1.22; *p* = 0.34; I² = 0%; four studies; 1,109 participants; very low certainty of evidence), LDL cholesterol (MD: −6.04; 95% CI: −12.42 to 0.35; *p* = 0.06; I² = 0%; four studies; 1,109 participants; low certainty of evidence), or triglycerides (MD: −6.03; 95% CI: −24.61 to 12.54; *p* = 0.52; I² = 0%; four studies; 1,109 participants; very low certainty of evidence). Although not statistically significant, the reduction in LDL cholesterol (*p* = 0.06) suggests a possible trend toward improvement, which should be explored in larger and more standardized trials.

**Fig. 3:**
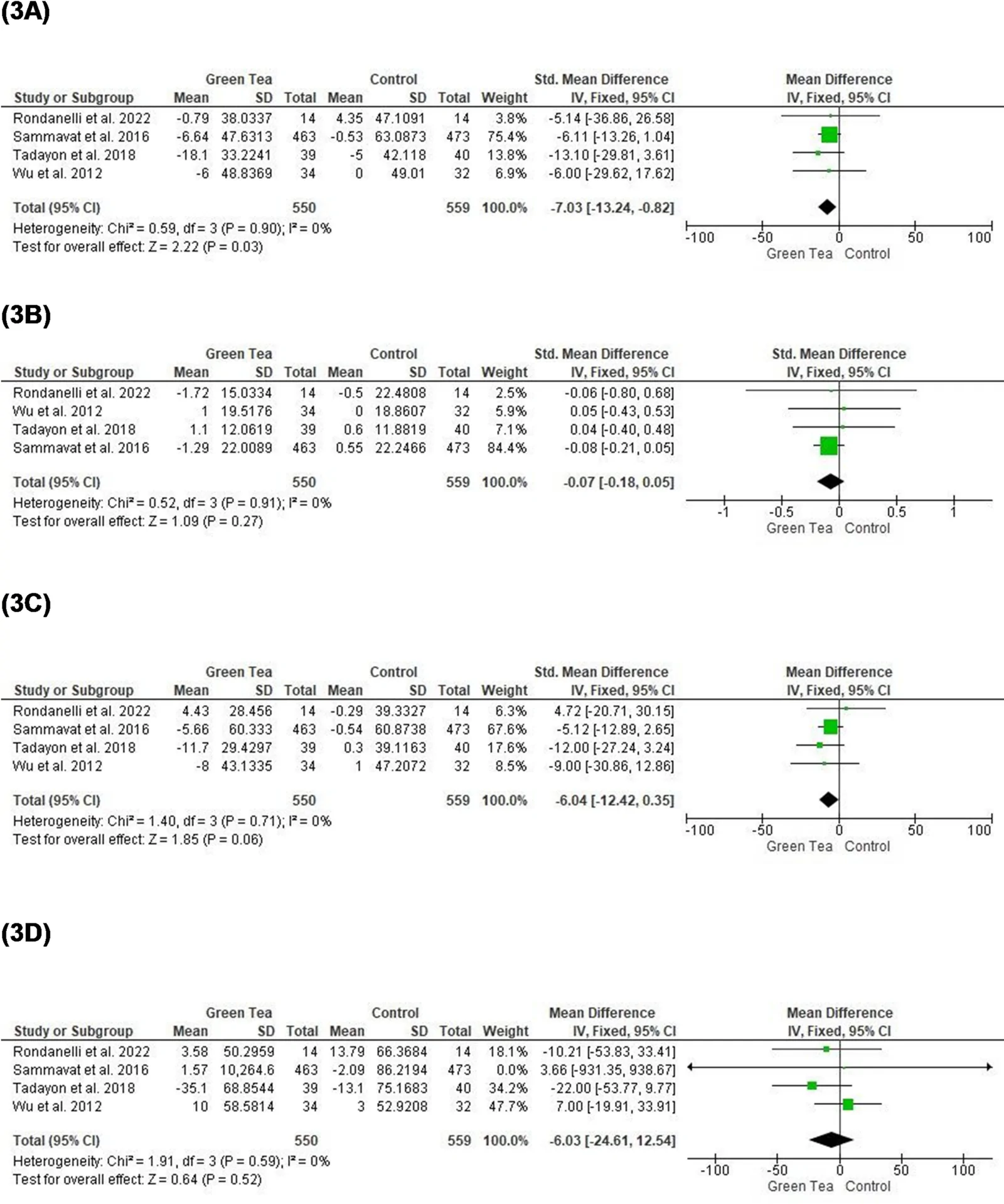
Meta-analysis of lipid profile outcomes: total cholesterol (**3A**), HDL cholesterol (**3B**), LDL cholesterol (**3C**), and triglycerides (**3D**). *Source*: Prepared by the authors (2026)

The meta-analyses of total cholesterol, HDL cholesterol, LDL cholesterol, and triglycerides are presented in Fig. 3A–D, respectively.

### Adipokines

**Fig. 4:**
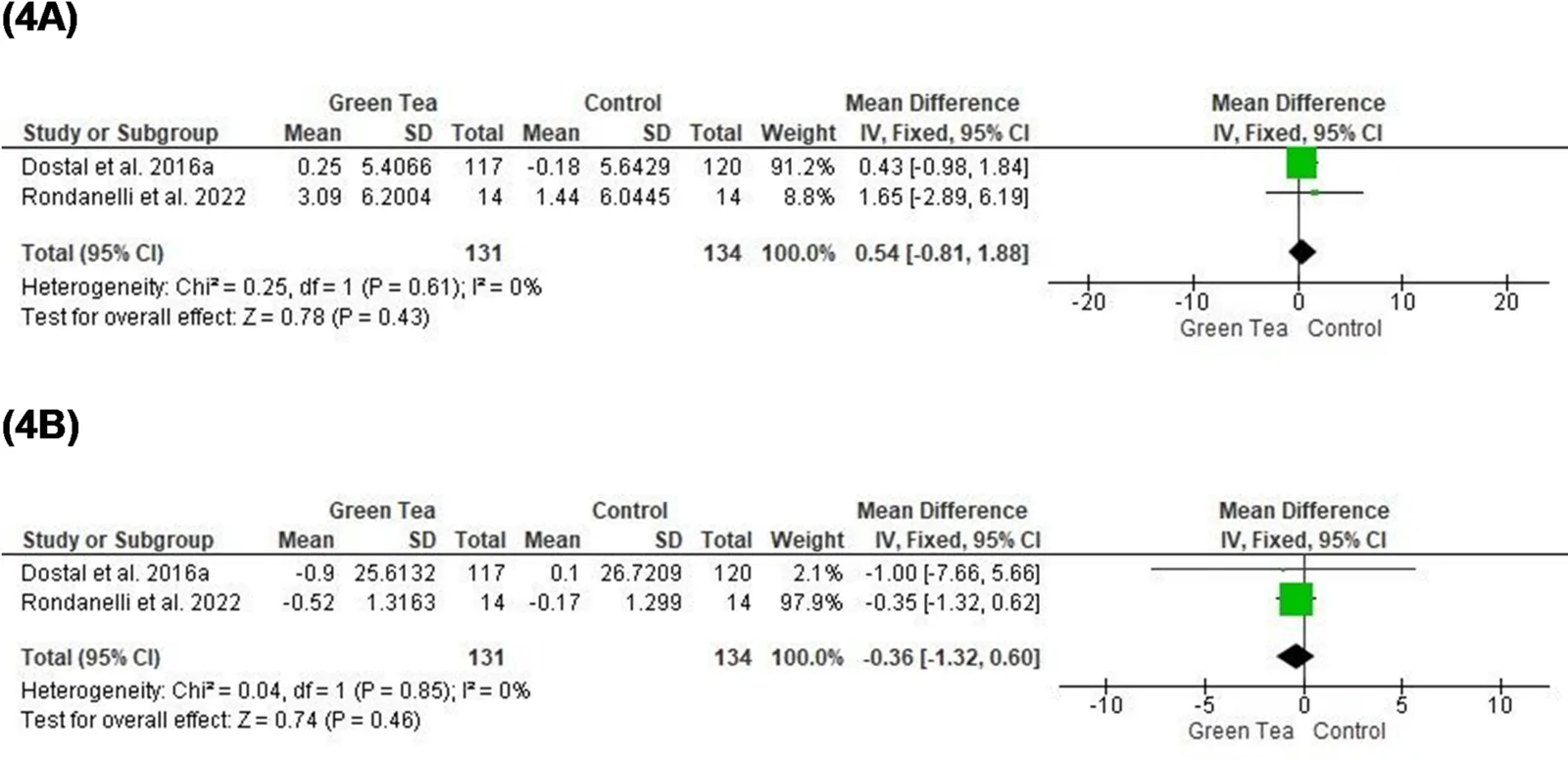
Meta-analysis of adipokine-related outcomes: adiponectin (**4A**) and leptin (**4B**). *Source*: Prepared by the authors (2026)

The adipokines adiponectin and leptin were evaluated in two of the included studies [12, 15], comprising a total of 131 participants in the green tea group and 134 in the control group. Meta-analysis of the study results showed no statistically significant differences between groups for adiponectin levels (MD: 0.54; 95% CI: −0.81 to 1.88; *p* = 0.43; I² = 0%; two studies; 265 participants; low certainty of evidence) or leptin levels (MD: −0.36; 95% CI: −1.32 to 0.60; *p* = 0.46; I² = 0%; two studies; 265 participants; low certainty of evidence), as shown in Fig. 4A, B, respectively.

### Glycemic and insulin outcomes

**Fig. 5:**
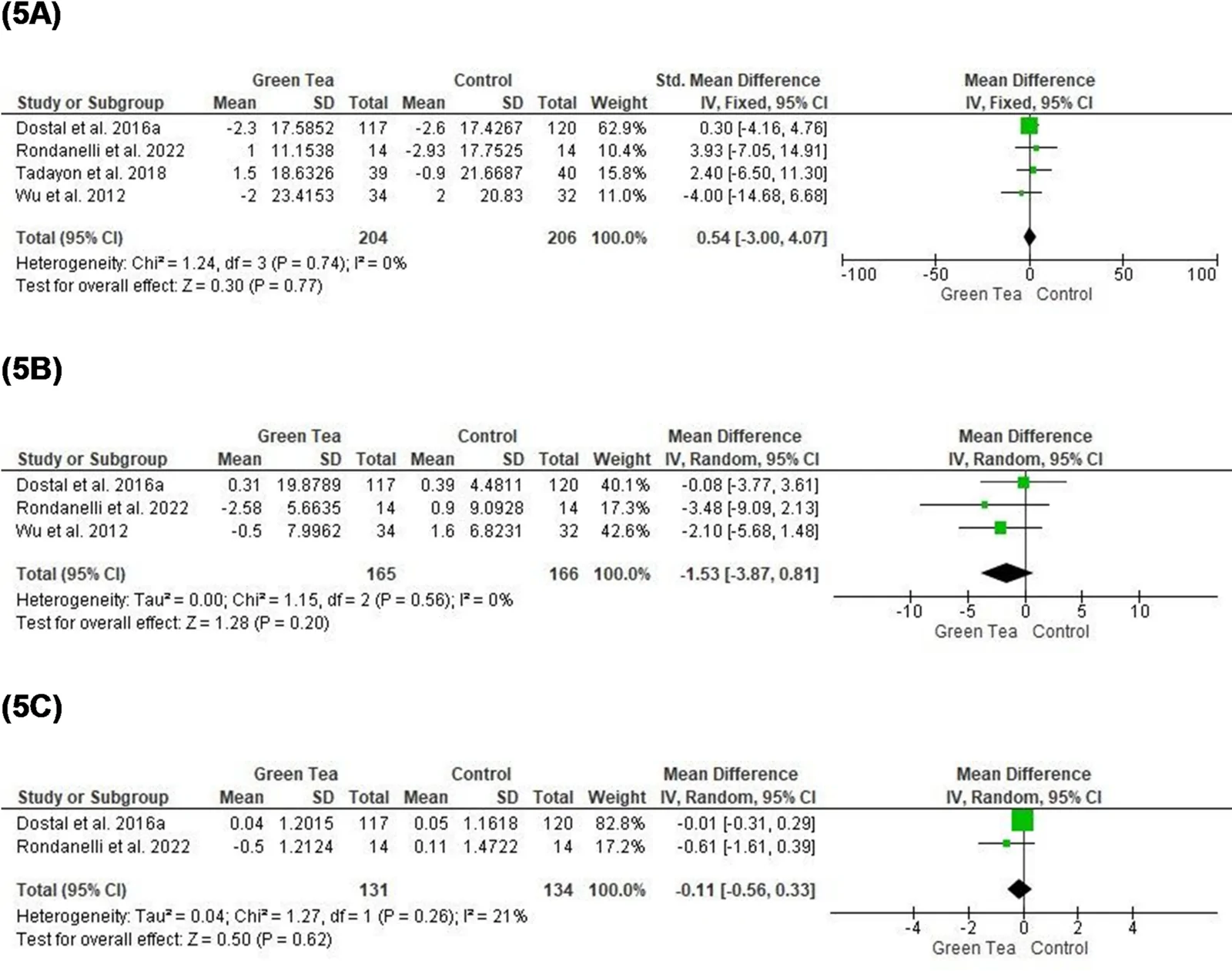
Meta-analysis of glycemic outcomes: blood glucose (**5A**), serum insulin (**5B**), and HOMA-IR (**5C**)

Reduction in blood glucose levels was evaluated in four of the included studies [13–15, 17], comprising a total of 204 participants in the green tea group and 206 in the control group. Between-group comparisons showed no statistically significant differences (MD: 0.54; 95% CI: −3.00 to 4.07; *p* = 0.77; I² = 0%; four studies; 410 participants; low certainty of evidence), as shown in Fig. 5A.

Serum insulin levels were assessed in three included studies [12, 15, 17], involving 165 participants in the intervention group and 166 in the control group. No statistically significant differences were observed between groups (MD: −1.53; 95% CI: −3.87 to 0.81; *p* = 0.20; I² = 0%; three studies; 331 participants; low certainty of evidence), as shown in Fig. 5B.

Two of the included studies reported HOMA-IR outcomes [12, 15], comprising 131 participants in the green tea group and 134 in the control group. Meta-analysis of these results revealed no statistically significant differences between groups, with low heterogeneity (MD: −0.11; 95% CI: −0.56 to 0.33; *p* = 0.62; I² = 21%; two studies; 265 participants; low certainty of evidence), as illustrated in Fig. 5C.

### Risk of bias of included studies

**Fig. 6:**
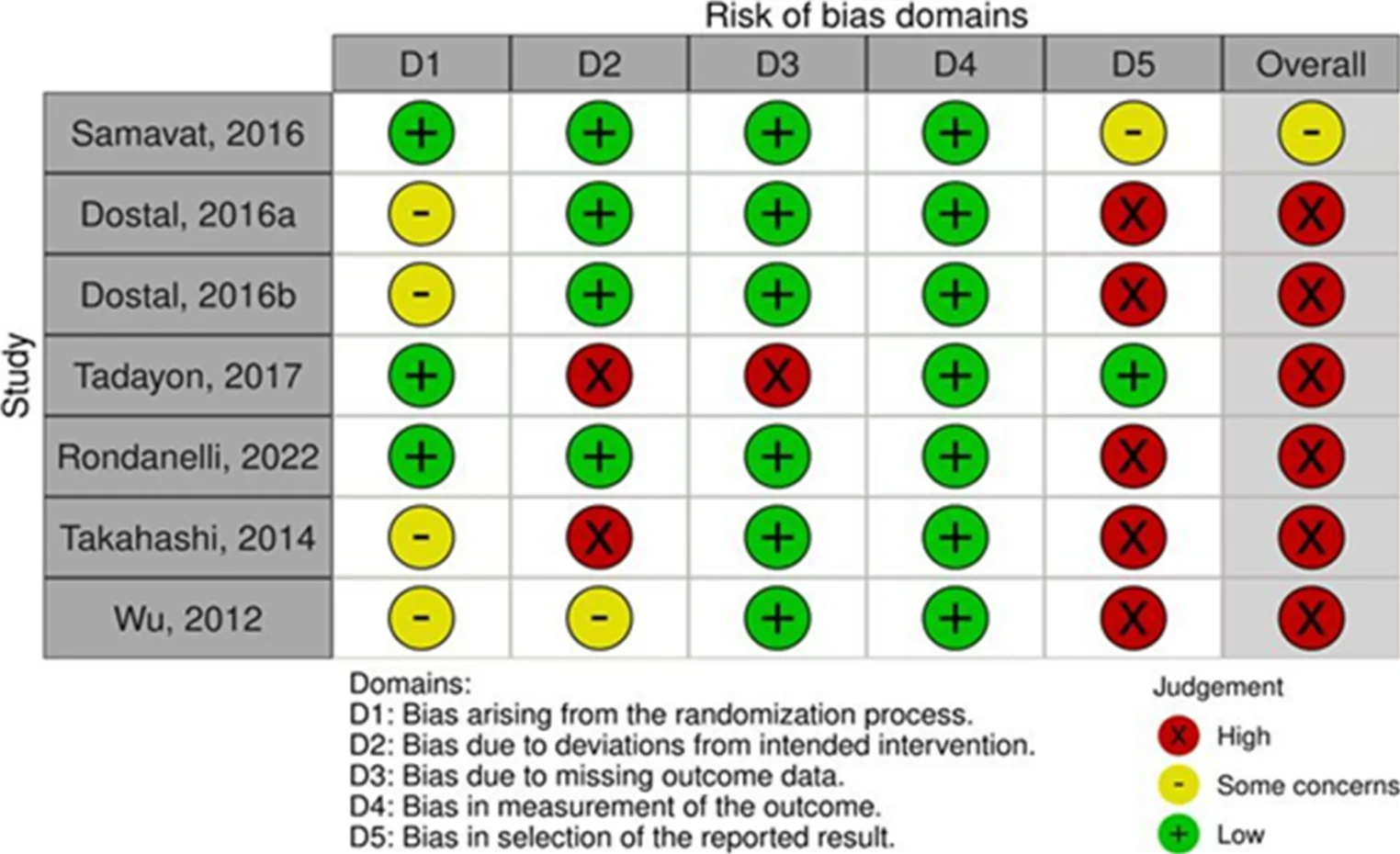
Risk of bias assessment of included studies. *Source*: Prepared by the authors (2026)

Risk of bias assessment of the included studies was conducted using the Cochrane Risk of Bias tool, version 2.0 (RoB 2.0). The results for each included study were assessed across the five domains and classified as “Low risk”, “Some concerns”, or “High risk”. The overall risk of bias judgment was based on the individual domain-level assessments. These evaluations are presented in Fig. 6.

Overall, most studies were judged to have a high overall risk of bias, with a predominance of either high risk or some concerns across multiple domains.

Regarding bias arising from the randomization process (Domain 1), four studies [12, 13, 16, 17] were judged as having some concerns, while the remaining studies were classified as low risk. In Domain 2 (bias due to deviations from intended interventions), only studies [14, 16] were judged as high risk. In study [14], the risk was attributed to the potential for unblinding and the use of an imperfect placebo, whereas in study [16], despite appropriate blinding, a dropout rate of 21% and an unclear analytical approach contributed to the high-risk judgment. All other studies were assessed as low risk in this domain.

For Domain 3 (bias due to missing outcome data), all studies except [14] were classified as low risk. Study [14] presented 21% missing data, with no clear evidence that the results were not biased by the missingness, raising concerns in this domain. The remaining studies demonstrated adequate data completeness.

In Domain 4 (bias in measurement of the outcome), no relevant issues were identified, and all studies were classified as low risk.

In Domain 5 (bias in selection of the reported result), study [14] was judged as low risk, whereas study [8] was classified as having some concerns, as it was a secondary analysis in which the analytical plan was not pre-specified in the original protocol. The remaining studies were classified as high risk in this domain, suggesting inconsistencies or lack of detailed pre-registration of outcomes. Trials [12, 13] relied on post hoc subgroup analyses using secondary outcomes and multiple subgroup comparisons that were likely not pre-specified in the original protocols, increasing the risk of selective reporting. In studies [15, 16], the absence of formal protocol registration, combined with a lack of clear outcome hierarchy and the presence of potential conflicts of interest, further reinforced concerns regarding reporting bias. In study [17], insufficiently detailed trial registration precluded verification of pre-specified outcomes, and the conduct of multiple additional analyses contributed to concerns about selective reporting. Consequently, all studies were classified as high risk in this domain.

In the overall risk of bias judgment, only study [8] was classified as having some concerns, whereas all remaining studies [12–17] were judged to be at high risk of bias.

### Quality of evidence analysis

The quality of evidence for the evaluated outcomes ranged from very low to low, according to GRADE criteria. For body weight, BMI, HDL cholesterol, and triglycerides, the certainty of evidence was rated as very low, with downgrading (− 3) due to high risk of bias (methodological limitations of the included studies) and imprecision (small sample sizes and wide confidence intervals).

For the lipid profile, the evidence supporting total cholesterol reduction was rated as low, with downgrading (− 2) primarily due to risk of bias. Similarly, the certainty of evidence for adipokines (adiponectin and leptin), glycemic markers (blood glucose, insulin, and HOMA-IR), and waist circumference was also rated as low. These findings underscore the need for caution in interpretation and highlight the importance of future well-designed randomized controlled trials with larger sample sizes and greater methodological rigor to confirm these results.

### Description of other evaluated outcomes

Due to methodological heterogeneity among the included studies, quantitative comparisons for several secondary outcomes, such as body composition, fat mass, resting energy expenditure, lipid oxidation, and inflammatory markers, were not feasible. For body composition outcomes specifically, pooling was limited by differences in assessment methods, particularly the use of dual-energy X-ray absorptiometry (DEXA) in Dostal et al. [13] and bioelectrical impedance analysis (BIA) in Rondanelli et al. [15].

The study by Dostal et al. [13] investigated body composition parameters, including fat mass, lean mass, visceral fat, and bone mineral density, and found no relevant differences after 12 months of supplementation. In contrast, the study by Rondanelli et al. [15] evaluated metabolic and inflammatory parameters and reported a significant increase in resting energy expenditure and lipid oxidation, along with a reduction in C-reactive protein (CRP), suggesting improvements in energy metabolism and inflammatory status in postmenopausal women. These findings are summarized in Table 2.

**Table 2:** Description of other outcomes evaluated

Author (year)	*N* (intervention/control)	Method	Outcome	Intervention (baseline → final)	Control (baseline → final)	*p*-value	Conclusion
Dostal et al. [13] (USA)	121 (61/60)	DEXA	Fat mass (kg)	32.2 ± 0.8 → 31.7 ± 0.8	30.9 ± 0.8 → 30.9 ± 0.8	0.40	No significant change
			Gynoid fat (%)	45.6 ± 0.6 → 45.5 ± 0.6	45.8 ± 0.6 → 45.7 ± 0.6	0.71	No significant change
			Visceral fat (kg)	1.06 ± 0.07 → 1.04 ± 0.07	0.88 ± 0.07 → 0.91 ± 0.07	0.11	No significant change
			Bone mineral density (g/cm²)	1.17 ± 0.01 → 1.17 ± 0.01	1.14 ± 0.01 → 1.14 ± 0.01	0.49	No significant change
Rondanelli et al. [15] (Italy)	28 (14/14)	BIA / Calorimetria indireta	Fat mass (kg)	t0:37.88 ± 6.01→t1: 36.47 ± 5.82→ t2:35.83 ± 5.48	t0:38.63 ± 9.23→t1: 38.02 ± 10.35→ t2:37.54 ± 10.61	0.22	No significant change
			Lean mass (kg)	t0:41.35 ± 3.58→t1: 41.37 ± 3.73→ t2:40.87 ± 3.33	t0:43.03 ± 4.0 →t1: 43.56 ± 3.7 → t2:43.016 ± 3.5	0.24	No significant change
			Visceral fat (kg)	t0:1.38 ± 0.42→t1:1.23 ± 0.48→ t2:1.21 ± 0.44	t0:1.39 ± 0.63→t1:1.37 ± 0.66→ t2:1.29 ± 0.83	0.73	No significant change
			RQ	t0:0.77 ± 0.06→t1:0.72 ± 0.05→ t2:0.71 ± 0.05	t0:0.79 ± 0.08→t1:0.79 ± 0.09→ t2:0.81 ± 0.05	0.009	Significant decrease (↑ increased fat oxidation)
			% LIP oxidation	t0:52.50 ± 18.28→t1: 78.86 ± 12.50 → t2: 78.79 ± 12.66	t0: 51.79 ± 16.57 →t1: 50.79 ± 16.22 → t2: 56.00 ± 18.83	0.0006	Significant increase in lipid oxidation
			REE (kcal/day)	t0: 1401.71 ± 202.88→t1: 1500.64 ± 229.12 → t2: 1537.21 ± 190.76	t0: 1396.57 ± 238.37 →t1: 1390.71 ± 201.04 → t2: 1365.64 ± 230.96	0.009	↑ Resting energy expenditure
			CRP (mg/L)	t0: 0.30 ± 0.22 →t1: - → t2: 0.16 ± 0.13	t0: 0.29 ± 0.22 →t1: - → t2: 0.29 ± 0.22	0.02	Significant reduction (↓ systemic inflammation)
			Norepinephrine (pg/mL)	t0: 509.50 ± 186.25 →t1: 571.50 ± 245.84 → t2: 627.64 ± 279.69	t0: 485.43 ± 185.13 →t1: 393.21 ± 145.13 → t2: 486.71 ± 128.97	0.22	No significant change
			Epinephrine (pg/mL)	t0: 31.07 ± 17.18 →t1: 31.29 ± 14.37→ t2: 36.93 ± 20.62	t0: 26.93 ± 11.49 →t1: 27.00 ± 19.91 → t2: 26.57 ± 19.25	0.66	No significant change

SD: Standard deviation; N: total number of participants; kg: kilograms; cm³: cubic centimeters; g/cm²: grams per square centimeter; pg/mL: picograms per milliliter; RCT: Randomized Clinical Trial; USA: United States of America; DEXA: Dual-Energy X-ray Absorptiometry; BIA: Bioelectrical Impedance Analysis; RQ: respiratory quotient; %LIP: percentage of lipid oxidation; REE: Resting Energy Expenditure; CRP: C-reactive protein; ↑: increase; ↓: reduction; t0: baseline; t1: after 30 days; t2: after 60 days; *p* < 0.05 was considered statistically significant

In addition, some included studies assessed complementary outcomes beyond the primary endpoints defined in this review. Wu et al. [17] evaluated hormonal variables, including sex hormone–binding globulin (SHBG), insulin-like growth factor 1 (IGF-1), and insulin-like growth factor–binding protein 3 (IGFBP-3), and observed no significant changes following green tea extract intervention. Takahashi et al. [16] assessed oxidative stress markers, including reactive oxygen metabolites (d-ROMs), hydrogen peroxide (H₂O₂), biological antioxidant potential (BAP), and thioredoxin (TRX), and reported an increase in TRX levels only in the intervention group, with no other significant differences. Although relevant, these parameters were not included in the quantitative analysis because they were not directly related to the predefined metabolic and anthropometric outcomes of the study protocol.

## Discussion

To our knowledge, this systematic review is the first to specifically evaluate the metabolic effects of green tea consumption in postmenopausal women. The findings indicate that green tea intake was associated with a modest but significant reduction in total cholesterol levels compared with placebo, while no significant effects were observed for the other evaluated outcomes, although a trend toward LDL reduction was identified.

These findings suggest that the metabolic effects of green tea in postmenopausal women may be attenuated compared to other populations, likely due to the hormonal and metabolic changes associated with estrogen deficiency. Previous systematic reviews and meta-analyses conducted in general adult populations have consistently demonstrated hypocholesterolemic effects of green tea. Since 2011, studies such as those by Kim et al. [18] and Zheng et al. [19] reported reductions in lipid markers, and more recently, Zamani et al. [20] observed significant reductions in total cholesterol with doses below 1,000 mg/day.

Although these findings are consistent with the reduction observed in the present study, it is important to highlight that these previous meta-analyses included heterogeneous populations with varying metabolic profiles. Therefore, extrapolation to postmenopausal women is limited, as this group presents a distinct metabolic environment characterized by estrogen deficiency, increased visceral adiposity, and alterations in lipid metabolism and insulin sensitivity.

Consistent with our findings, Xu et al. [21] also reported reductions in total and LDL cholesterol, with no significant effects on HDL cholesterol or triglycerides. However, the variability in results across studies suggests that metabolic responses may be influenced by baseline metabolic status, intervention characteristics, and population-specific factors.

Taken together, these findings indicate that the present study refines existing evidence by demonstrating that the metabolic effects of green tea, although consistent with previous meta-analyses, may be attenuated in postmenopausal women, reinforcing the importance of population-specific analyses. Recent evidence from emerging systematic reviews has also reinforced the potential role of green tea in modulating cardiometabolic risk, although with heterogeneous findings depending on population and intervention characteristics [22–24].

One important factor contributing to this variability is the dosage of epigallocatechin-3-gallate (EGCG), the main bioactive compound in green tea. Evidence suggests a dose-dependent effect, with higher doses and longer intervention durations associated with more favorable metabolic outcomes [25]. However, the wide variation in EGCG doses across studies limits the identification of a minimum effective dose. Although a clear minimum effective dose cannot be definitively established due to heterogeneity across studies, trials reporting favorable metabolic effects generally used EGCG doses above 400 mg/day. This suggests that lower doses may be insufficient to induce clinically meaningful changes, although this hypothesis requires confirmation in well-designed dose–response trials.

In addition to dosage, the formulation of green tea appears to play a significant role. Xu et al. [21] reported that decaffeinated green tea tends to exert smaller or inconsistent effects compared with caffeinated preparations. Similarly, studies included in the present review that used decaffeinated formulations [8, 12, 13] showed fewer significant effects. This finding suggests a potential synergistic interaction between catechins and caffeine, possibly mediated by increased thermogenesis and fat oxidation [21]. This variability in formulation represents a key limitation, as differences in bioavailability and metabolic effects between extracts and beverages, as well as between caffeinated and decaffeinated preparations, may have significantly influenced the outcomes.

From a mechanistic perspective, the biological effects of green tea are largely attributed to EGCG, which acts through multiple pathways, including activation of AMP-activated protein kinase (AMPK), stimulation of fatty acid oxidation, reduction of lipogenesis, inhibition of adipocyte differentiation, and modulation of the gut microbiota [26].

Additionally, catechins exert antioxidant and anti-inflammatory effects that may contribute to improved endothelial function and lipid metabolism [8, 25]. Supporting these mechanisms, Chen et al. [27] demonstrated that high-dose EGCG supplementation (856.8 mg/day) resulted in reductions in body weight, BMI, and waist circumference, along with improvements in lipid profile and adiponectin levels.

Despite the low statistical heterogeneity observed across most pooled analyses, substantial clinical heterogeneity was present. The included studies differed considerably in intervention type (extract vs. beverage), EGCG dosage, duration of supplementation, and participant metabolic profiles. These differences likely influenced the magnitude and direction of the observed effects and may partially explain the inconsistency of findings across studies.

These findings also suggest that baseline metabolic status may modulate responsiveness to green tea interventions, as supported by evidence from Colonetti et al. [28], who reported reductions in body weight in women with polycystic ovary syndrome following green tea supplementation. Although this effect was not observed in the present study, it suggests that individuals with greater metabolic dysfunction may derive more pronounced benefits.

Accordingly, the modulatory effects of green tea on energy metabolism appear to occur gradually and depend on both EGCG concentration and duration of supplementation [29–31].

Interindividual variability may also contribute to inconsistent findings across studies. Factors such as ethnicity, caffeine metabolism, and catechol-O-methyltransferase (COMT) activity influence catechin bioavailability and metabolic responses [12, 21].

The clinical relevance of the observed reduction in total cholesterol should also be interpreted with caution. Although statistically significant, the magnitude of reduction (approximately − 7 mg/dL) is modest. Moreover, the significant reduction in total cholesterol should be interpreted in light of the dominance of the Samavat et al. [8] trial in the pooled analysis. This limits the robustness of the finding and suggests that the overall effect may be less stable than indicated by the low statistical heterogeneity. However, even small reductions may contribute to cumulative cardiovascular risk reduction when integrated into broader lifestyle interventions, particularly in populations at increased baseline risk.

Regarding safety, most included trials reported good tolerability. However, one study reported increased liver enzyme levels in participants receiving green tea extract [12]. Although green tea as a beverage is generally considered safe, concentrated extracts, particularly those with high EGCG content, have been associated with dose-dependent hepatotoxicity [32, 33].

Therefore, caution is warranted when using high-dose supplements, and monitoring of hepatic function may be advisable.

Finally, the overall certainty of evidence was rated as low to very low for most outcomes, primarily due to high risk of bias, small sample sizes, and imprecision. Importantly, the high risk of bias observed in most included studies substantially limits the reliability of the findings. This methodological limitation may have influenced the direction and magnitude of the observed effects, reducing confidence in the findings and reinforcing the need for cautious interpretation.

## Conclusion

This systematic review provides evidence that green tea consumption may lead to modest reductions in total cholesterol levels in postmenopausal women, although no significant effects were observed for other metabolic outcomes. Future high-quality randomized controlled trials with standardized dosage, formulation, and duration are needed to confirm these findings and support the safe and effective clinical use of green tea as an adjunctive strategy. Although green tea appears to be a promising adjunctive strategy, high-dose concentrated extracts should be used with caution, and hepatic function monitoring may be warranted in clinical practice.

## Electronic Supplementary Material

Below is the link to the electronic supplementary material.

Supplementary Material 1

## Data Availability

The datasets used during the current study are available from the corresponding author on reasonable request.
