# Correction: Effects of green tea use on the metabolic profile of postmenopausal women: systematic review and meta-analysis

**DOI:** 10.1007/s00394-026-04125-1

**Published:** 2026-10-27

**Authors:** Isabella Heloísa Rodrigues Zago, Laura Colonetti, Eduarda Letícia Balbinot, Igor Specht Taschetto, Antonio José Grande, Maria Inês da Rosa, Tamy Colonetti

**Affiliations:** 1 School of Medicine, Universidade do Extremo Sul Catarinense (UNESC), Criciúma, SC Brazil; 2 Laboratory of Translational Biomedicine, Universidade do Extremo Sul Catarinense, Criciúma, SC Brazil; 3 Laboratory of Evidence-Based Practice, Universidade Estadual de Mato Grosso do Sul, Campo Grande, MS Brazil

**Correction to: European Journal of Nutrition (2026) 65:150** **https://doi.org/10.1007/s00394-026-04005-8**

In the original version of this article, the total participant count was incorrect in the section ‘’Description of included studies’’.

In the section ‘’Description of included studies’’, first sentence which previously read as 

The seven included studies were published between January 2012 and January 2023 and involved a total of 549 participants, all of whom were women, with a mean age ranging from 49 to 67 years.

but the sentence should have read as

The seven included studies were published between January 2012 and January 2023 and involved a total of 1,168 unique participants, all of whom were women, with a mean age ranging from 49 to 67 years.

