# Supplementary material for: Effects of green tea use on the metabolic profile of postmenopausal women: systematic review and meta-analysis
Source: Eur J Nutr. 2026 Jun 2;65(4):150. doi: 10.1007/s00394-026-04005-8 (PMC13230310; doi:10.1007/s00394-026-04005-8)
Supplement: Supplementary file 1 — Supplementary Material 1 [file 394_2026_4005_MOESM1_ESM.docx]

**EFFECTS OF GREEN TEA USE ON THE METABOLIC PROFILE OF POSTMENOPAUSAL WOMEN: SYSTEMATIC REVIEW AND META-ANALYSIS**

**European Journal of Nutrition**

Isabella H. Rodrigues Zago¹, Laura Colonetti^2^, Eduarda Letícia Balbinot¹, Igor Specht Taschetto¹, Antônio José Grande³, Maria Inês da Rosa^2^, Tamy Colonetti²

¹ School of Medicine, Universidade do Extremo Sul Catarinense, Criciúma, Santa Catarina, Brazil

² Laboratory of Translational Biomedicine, Universidade do Extremo Sul Catarinense, Criciúma, Santa Catarina, Brazil.

³ Laboratory of evidence-based practice, Universidade Estadual de Mato Grosso do Sul, Campo Grande, MS, Brazil.

Corresponding Author: Tamy Colonetti.

School of Medicine, Universidade do Extremo Sul Catarinense (UNESC), Criciúma, Santa Catarina, Brazil. Email: tamycolonetti@unesc.net

**Supplementary material**

**Supplementary Material 1.** Search Strategies

| **MEDLINE (via PubMed)** |
| --- |
| ("Tea"[Mesh] OR "Green Tea"[Mesh] OR "Camellia sinensis"[All Fields] OR "green tea"[All Fields] OR "green tea extract"[All Fields] OR "green tea catechins"[All Fields] OR "epigallocatechin gallate"[All Fields] OR "EGCG"[All Fields] OR "Polyphenon E"[All Fields])  AND  ("Menopause"[Mesh] OR "Postmenopause"[Mesh] OR "postmenopausal"[All Fields] OR "postmenopause"[All Fields]) |
| **Embase (Elsevier)** |
| ('green tea'/exp OR 'green tea' OR 'green tea extract' OR 'green tea catechin' OR 'epigallocatechin gallate' OR egcg OR 'camellia sinensis')  AND  ('menopause'/exp OR 'postmenopause'/exp OR menopause OR postmenopause OR postmenopausal) |
| **Web of Science** |
| TS=("green tea" OR "green tea extract" OR "green tea catechins" OR "epigallocatechin gallate" OR EGCG OR "camellia sinensis")  AND  TS=(menopause OR postmenopause OR postmenopausal) |
| **Cochrane Library** |
| ("green tea" OR "green tea extract" OR "green tea catechins" OR "epigallocatechin gallate" OR EGCG)  AND  (menopause OR postmenopause OR postmenopausal) |
| **LILACS (BVS)** |
| ("green tea" OR "chá verde" OR "extrato de chá verde" OR "catequinas do chá verde" OR "epigalocatequina galato" OR EGCG)  AND  (menopausa OR pós-menopausa OR postmenopause OR postmenopausal) |
